# Synthetic extreme environments: overlooked sources of potential biotechnologically relevant microorganisms

**DOI:** 10.1111/1751-7915.12602

**Published:** 2017-02-22

**Authors:** Timothy Sibanda, Ramganesh Selvarajan, Memory Tekere

**Affiliations:** ^1^ Department of Environmental SciencesCollege of Agriculture and Environmental ScienceUNISA Florida CampusPO Box X6Florida1709South Africa

## Abstract

Synthetic extreme environments like carwash effluent tanks and drains are potential sources of biotechnologically important microorganisms and molecules which have, however, remained unexplored. Using culture‐ and molecular‐based methods, a total of 17 bacterial isolates belonging to the genera *Shewanella*,* Proteus*,* Paenibacillus, Enterobacter* and *Citrobacter*,* Aeromonas*,* Pseudomonas* and *Pantoea* were identified. Hydrocarbon utilization and enzyme production screening assays showed that *Aeromonas* sp. CAC11, *Paenibacillus* sp. CAC12 and *Paenibacillus* sp. CAC13 and *Citrobacter* sp. PCW7 were able to degrade benzanthracene, naphthalene and diesel oil, *Paenibacillus* sp. CAC12 and *Paenibacillus* sp. CAC13 could produce cellulase enzyme, while *Proteus* sp. BPS2, *Pseudomonas* sp. SAS8 and *Proteus* sp. CAL3 could produce lipase. GC‐MS analysis of bacterial secondary metabolites resulted in identification of 107 different compounds produced by *Proteus* sp. BPS2, *Paenibacillus* sp. CAC12, *Pseudomonas* sp. SAS8, *Proteus* sp. CAL3 and *Paenibacillus* sp. CAC13. Most of the compounds identified by both GC‐MS and LC‐MS have previously been determined to have antibacterial, antifungal and/or anticancer properties. Further, microbial metabolites which have previously been known to be produced only by plants or microorganisms found in natural extreme environments were also identified in this study. This research has revealed the immense bioresource potential of microorganisms inhabiting synthetic extreme environments.

## Introduction

Microbial life has been found to adapt to and thrive in extreme conditions ranging from extremely low water availability, intense solar radiation, high salinity and extreme temperatures, pH and pressure (Rampelotto, 2013; Azua‐Bustos and González‐Silva, 2014). Natural extreme environments can be defined as habitats that experience steady or fluctuating exposure to one or more environmental factors such as salinity, osmolarity, desiccation, UV radiation, barometric pressure, pH and temperature (Seufferheld *et al*., 2008). Some extreme environments are, however, synthetic (Maes *et al*., 2016) and may be created by a sustained and consistent discharge of pollutants (like acid mine drainage, untreated municipal sewage effluents and industrial effluent discharges among others) into the environment (Selbmann *et al*., 2013), making it very selective and often hostile to major life forms.

Microorganisms that inhabit extreme environments are referred to as extremophiles and are broadly classified into extremophilic organisms (which require one or more extreme conditions to grow), and extremotolerant organisms (which can tolerate extreme values of one or more physicochemical parameters though growing optimally at normal conditions) (Rampelotto, 2013). Extremophiles from a wide range of natural extreme environments have been characterized, and indeed, a lot of biotechnological applications have been made stemming from their secondary metabolites (Seufferheld *et al*., 2008; Azua‐Bustos and González‐Silva, 2014). Relatively few researchers (Chronakova *et al*., 2010; Perks, 2011) have characterized microorganisms from synthetic extreme environments with the aim to biotechnologically exploit them. Therefore, the varieties of microorganisms in synthetic extreme environments, together with the molecular mechanisms they have evolved to cope with abiotic stresses in their environments, still need to be elucidated.

The current challenge besetting the study of biological molecules produced by extremophiles is that their potential applications may not be well known, or even unknown (Azua‐Bustos and González‐Silva, 2014). This, however, offers an immense potential for future development. To this end, evidence from past research suggests biotechnologically significant roles ranging from antimicrobials to anticancer molecules (Perks, 2011). Among the most explored applications of extremophiles are their production of enzymes (also referred to as extremozymes) (Demirjian *et al*., 2001; Enache and Kamekura, 2010), fatty acids and proteins (Cavicchioli *et al*., 2002; Reed *et al*., 2013), antibiotics or biomedicines (Azua‐Bustos and González‐Silva, 2014), and the direct application of cultures in chemical processes like desulfurication of flue gases and in biohydrometallurgical processes (Huber and Stetter, 1998). Biohydrometallurgical processes include, but are not limited to, microbial mining of precious (and rare) metals, oil recovery, bioleaching and water treatment.

Among microbial groups most commonly found in extreme environments, actinobacteria are arguably the richest source of small molecule diversity, with widespread global and environmental dispersal (Jami *et al*., 2015; Kuang *et al*., 2015; Ettoumi *et al*., 2016). Synthetic extreme environments like carwash effluent tanks and drains remain unexplored, and their potential as a source of biotechnologically important molecules remains unknown. While synthetic extreme environments are (almost all) seriously polluted sites which are in need of remediation, they may conversely be harbouring life‐saving agents and other biotechnologically important molecules. As the survival strategies of extremophiles are often novel and unique (Seufferheld *et al*., 2008), the necessity for microbial cell components to adapt to extreme environments (natural or synthetic) implies that a broad range of cellular products (genes and metabolites) are available for biotechnological applications.

While there exists no previous literature records elucidating the microbial composition of carwash effluents, Tekere *et al*. (2016) characterized the physicochemical properties of carwash effluents which they have shown to have high levels of, among other pollutants, petroleum hydrocarbons, polycyclic aromatic hydrocarbons (PAHs), phenols and heavy metals. Further, these effluents were shown to be toxic not only to animals and plants but were also found to inhibit light production by the bacterium *Vibrio fischeri*. PAHs are the most common environmental pollutants which enter both the terrestrial and aquatic environments in large volumes, posing a great hazard to ecosystems (Bayat *et al*., 2015). Research has shown that in the short to medium term, contamination of environmental media with heavy metals results in reduced microbial biomass, coupled with reduced enzyme activity (Wang *et al*., 2007; Akmal, 2009). In the long term, environmental media contaminated with PAHs showed reduced microbial biodiversity and species richness index (Markowicz *et al*., 2016). However, the same study by Markowicz *et al*. (2016) showed that while soils contaminated with heavy metals had reduced microbial activity and changes in microbial community structure, a high microbial evenness index was observed. This suggests that some populations of the microbial community are ‘enriched’ by the presence of the very same pollutants which diminish the population sizes of the less tolerant microorganisms. Assessment of the impact of long‐term diesel contamination on soil microbial community structure, for example, showed both a decrease in the relative abundance of certain bacterial phyla and an increase in the relative abundance of other phyla (Sutton *et al*., 2013). The microbes that undergo natural attenuation to adapt to extreme environments could be sources of biotechnologically indispensable molecules with a wide range of applications ranging from bioremediation of polluted sites to producing industrially relevant enzymes and other products. The aim of this study therefore was to characterize bacterial isolates from carwash effluent samples and to determine their ability to produce some target enzymes as well as other secondary metabolites, previously known or unknown, and create a metabolic fingerprint.

## Experimental procedures

### Description of study area

**Figure 1:**
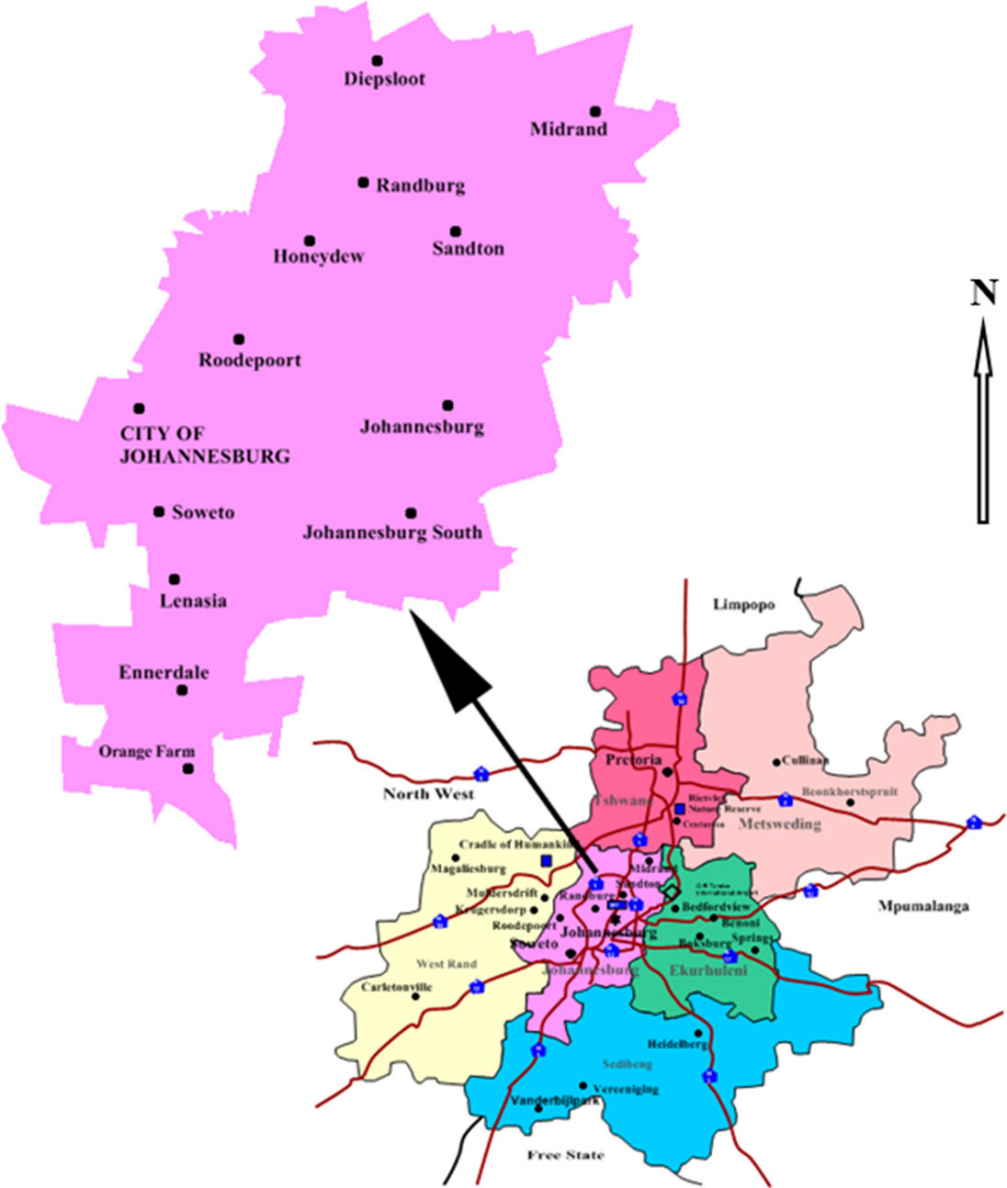
Map of Gauteng Province in South Africa showing the Johannesburg Metropolitan Municipality demarcation (not drawn to scale).

Carwash effluent samples were obtained from six carwash stations in the Johannesburg Metropolitan Municipality located in Gauteng Province in South Africa. Three carwash stations were located in Johannesburg City, while the other three were in the affluent business area, Sandton City (Fig. 1).

For confidentiality purposes, the exact locations and/or real names of the carwashes are not mentioned. However, the sampling stations were code‐named as BPS, CAL and SAS (Johannesburg), and CAC, PCW and TCWPM (Sandton).

### Sample collection, bacterial isolation and characterization

One‐litre (1 l) grab samples were obtained from the onsite treatment facility of each carwash using sterile plastic sampling bottles. The samples were immediately chilled by placing them in cooler boxes containing ice and transported to the laboratory at UNISA Florida Campus for analysis within 6 h of collection. Once in the laboratory, the samples were shaken to ensure homogeneity after which 100 μl aliquots of each sample were spread plated in triplicate onto freshly prepared nutrient agar (Sigma Aldrich, Pretoria, South Africa). The plates were incubated at 30°C for 48 h. The resultant (mixed) cultures were separated and purified by subculturing onto nutrient agar plates until axenic cultures were obtained. DNA was then isolated from each culture using a Quick *g*‐DNA extraction kit (Zymo Research, Irvine, CA 92614, U.S.A.) followed by PCR amplification using 16S universal bacterial primers (27 F and 1492 R). The PCR amplicons were confirmed by gel electrophoresis and then sent to Inqaba Biotec (Pretoria, South Africa) for sequence analysis. The resultant sequence chromatograms were manually edited with Chromas software v2.6.1 (Technelysium Pty Ltd., South Brisbane, Qld, Australia) and subjected to BLAST analysis to compare the identity of the isolates. The sequences were then used for phylogenetic analysis using the molecular evolutionary genetic analysis v6.0 (MEGA6) software (Tamura *et al*., 2013) using an alignment created with SINA Aligner. A phylogenetic tree was constructed using maximum‐likelihood analysis with kimura 2‐parameter model and 500 times of bootstrap replications. Finally, the sequences were submitted to Gene bank to obtain the accession numbers.

### Screening for hydrocarbon utilization

The isolates were tested for the capacity to degrade hydrocarbons following a modified protocol previously described by Um *et al*. (2010). Minimal salt medium (MSM) containing in g/l: NaCl 5.0, K_2_HPO_4_ 1.0, NH_4_H_2_PO_4_ 1.0, (NH4)_2_SO_4_ 1.0, MgSO_4_. 7H_2_O 0.2 and KNO_3_ 3.0 supplemented with 1.25% (w/v) of agar, was autoclaved for 15 min and poured out onto Petri plates as ‘bottom agar’. This bottom agar was then overlaid with 100 μl of a polycyclic aromatic hydrocarbon (PAH) solution prepared by dissolving 10 mg/50 ml naphthalene and 10 mg/10 ml benzanthracene (Sigma, Pretoria, South Africa) in methanol. The PAH solution was evenly spread over the agar surface using sterile disposable spreaders and the plates were left in the lamina flow for the solvent to evaporate, leaving behind a visible thin white layer of PAHs on the surface of the bottom agar. To inoculate the bottom agar, 100 μl of each bacterial isolate was mixed with 900 μl of 35–40°C molten ‘top agar’ medium containing 0.5% agar and same mineral composition as the bottom agar and immediately poured on Petri plates containing bottom agar. The plates were then swirled gently to spread molten top agar medium over bottom agar, which resulted in the white layer of PAHs to move to the surface of the top agar medium upon solidifying. The inoculated plates were incubated at 25°C and examined daily for the presence of growth and clear zones.

The isolates which were able to degrade PAHs (seen by formation of clear halos colonies) were selected for hydrocarbon‐degrading assays following the method of Oliveira *et al*. (2012). The isolates were inoculated into 20‐ml tubes containing 10 ml of fermentation broth made up of minimal salt medium supplemented with 10 g/l of glucose and 1.0 g/L of yeast extract and incubated at 25°C for 72 h with shaking at 120 r.p.m. After incubation, the inoculum was centrifuged at 10 000 r.p.m. for 5 min at 10°C. The cell pellet was resuspended in phosphate buffer (pH 7) and again centrifuged at 10 000 r.p.m. for 5 min at 10°C, discarding the supernatant. To remove all culture medium residues, this step was performed twice. After the final centrifugation, the isolates were resuspended in phosphate buffer and the OD adjusted to McFarland 0.5. The assay was carried out in a sterile 96‐well microtitre plate by adding 20 μl of the isolate suspension in triplicate, to a row of wells containing 168 μl of minimal salt medium, 12 μl of 2,6‐dichlrophenolindophenol (DCPIP) and 2 μl of diesel oil. Each isolate was inoculated into a row of wells containing three different hydrocarbons, the other two being naphthalene and benzanthracene. In addition to the test wells containing isolates and different hydrocarbons, the experiment had a positive control produced by mixing 148 μl of minimal medium with 12 μl of DCPIP solution, 20 μl of 10% glucose solution and 20 μl of the *Pseudomonas* cell suspension; and a negative control consisting of 168 μl of minimal medium, 20 μl of the isolate suspension and 12 μl of DCPIP solution. The plates were incubated at 30°C, and the readings were taken after 24, 48 and 72 h of incubation using a spectrophotometer at 600 nm. The percentage reduction in DCPIP was obtained using the following equation:

$$\%\text{DCPIP reduction}=\frac{\text{0h}\;{\text{OD}}_{600}-\text{Yh}\;{\text{OD}}_{600}}{\text{0h}\;{\text{OD}}_{600}}\times 100\%,$$

where the value of Y was, sequentially, 24, 48 and 72 h.

### Screening for enzyme production (lipase and cellulase)

Production of lipase was determined by growing the bacterial isolates in rhodamine–olive oil–agar medium following the method of Kumar *et al*. (2012) with little modifications. The agar medium contained, in g/l, agar–agar 20, MgSO_4_ 0.2, CaCl_2_ 0.02, KPO_4_ 1.0, K_2_PO_4_ 1.0, NH_4_NO_3_ 1.0, FeCl_3_ 1.0 and yeast extract 5.0. The medium was adjusted to pH 7.0, autoclaved and cooled to about 50°C after which 31.25 ml of olive oil and 10 ml of rhodamine B solution (1.0 mg/ml distilled water and sterilized by filtration) were added with vigorous stirring. It was then poured into Petri plates under aseptic conditions and allowed to solidify. The plates were inoculated by smearing buttons of bacterial cultures on the agar surface. After incubation of the plates for 48 h at 30°C, they were viewed under UV irradiation and lipase‐producing strains were identified by formation of orange fluorescent halos around bacterial colonies due to the hydrolysis of substrate.

For cellulase screening, agar medium containing 0.2% (w/v) carboxymethylcellulose sodium salt (CMC), 1% agar and minimal salt medium was prepared and poured into Petri plates. Spot inoculation was then performed using axenic cultures of the test isolates followed by incubation at 30°C for 48 h. Hydrolysis zones were visualized by flooding the plates with 0.1% Congo red stain (Glass World, Johannesburg, South Africa) and allowing to stand for 15 min followed by destaining with 1 M NaCl.

### Analysis of bacterial secondary metabolites

Bacterial isolates which were positive for enzyme production screens were grown in fermentation media consisting of minimal salt medium supplemented with olive oil and CMC‐Na salt for 7 days in a shaking incubator at 30°C. After incubation, cell debris was separated from the supernatant under high‐speed centrifugation at 4°C. The supernatant was subjected to solvent extraction of secondary metabolites. Briefly, the supernatant was a mixture of chloroform and methanol (1:1, v/v) and shaken for 12 h at 120 r.p.m. and 25°C. The ethyl acetate fraction was then separated from the aqueous fraction using a separating funnel. The solvent fraction was evaporated to dryness *in vacuo* at 80°C, and the residue was reconstituted in a mixture of 1:1 (v:v) acetonitrile and hexane. The acetonitrile fraction was used to analyse for the polar secondary metabolites using LC‐MS, while the hexane fraction was used to analyse for non‐polar, volatile compounds using GC‐MS.

For GC analysis, ionization energy was set at 70 eV using He as the carrier gas. The flow rate was set at 1 ml/min, injection volume at 2 μl, split ratio at 10:1, injection temperature at 250°C, ion source temperature at 200°C and oven temperature at 110°C (isothermal at 2 min) with increase of 10°C/min to 200°C then 5°C/min to 280°C (with 9 min isothermal at 280°C). An HP‐5MS fused silica capillary column (30 m, 0.25 mm i.d., 0.25‐μm film, cross‐linked to 5% phenyl methyl siloxane stationary phase) was used. For MS, ionization energy was set at 70 eV, scan interval at 0.5 s, fragments at 45–450 kD and solvent delay at 0–2 min. The identification of compounds was based on comparison of their mass spectra with the National Institute of Standards and Technology (NIST 2005) library.

LC‐MS was performed using an ultra high‐performance liquid chromatography–mass spectrophotometer (Compass otofSeries 1.9, Bruker Instrument: ImpactII) system. For LC analysis, the column was Acquity UPLC BEH C18 1.7 μm, diameter 2.1 × 100 mm (Miscrosep Waters, Johannesburg, South Africa), and the solvents were 0.1% formic acid (FA) in water and 0.1% FA in acetonitrile. The column flow was set at 0.3 ml/min, column oven temp at 35°C and draw speed at 2 μl/s with a total injection volume of 2 μl. Mass spectrometer (MS) conditions were set at a mass range of 50–1600 m/z, capillary 4500 v, dry gas 8 l/m, gas temperature 220°C, ion energy 4.0 eV, collision energy 7.0 eV, cycle time 0.5 s. Data analysis was performed using the bruker Software (Bruker Compass DataAnalysis 4.3; Bruker Daltonik GmbH 2014, Bremen, Germany).

## Results and discussion

### Isolation and characterization of bacterial isolates in carwash samples

**Figure 2:**
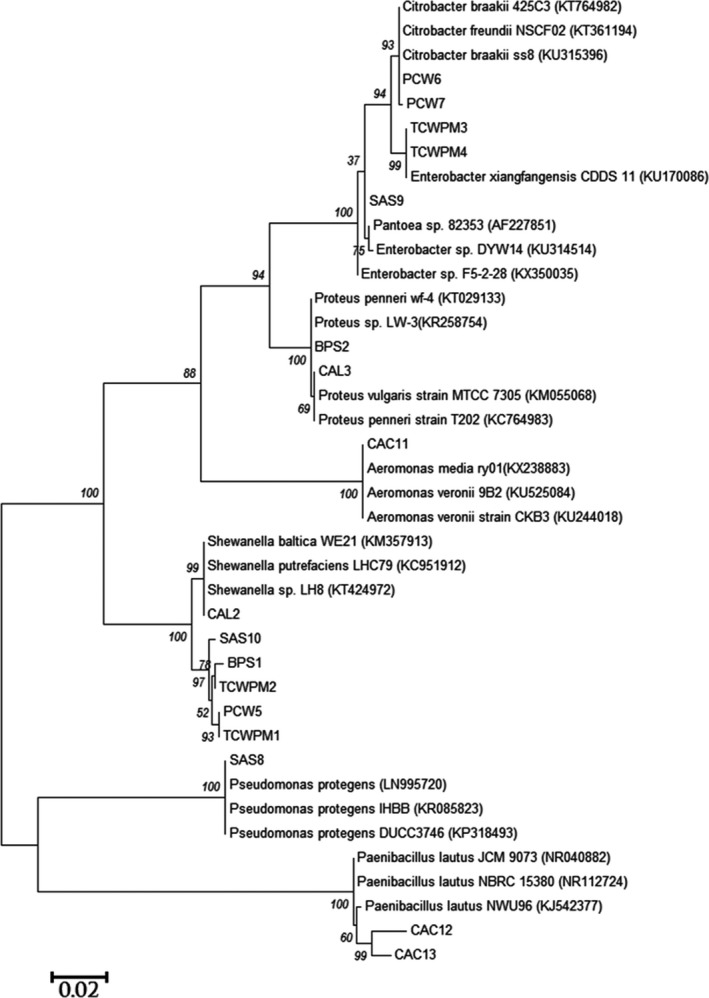
Phylogenetic tree based on 16S rDNA gene sequences obtained by the maximum‐likelihood method showing the phylogenetic relationship among the 17 bacterial isolates of this study (code names) and related bacteria.

A total of 17 different isolates were obtained from all the six carwash sampling sites. Phylogenetic comparison of PCR‐amplified 16S rDNA sequence data of each isolate with the database of the databases of known species using the NCBI server revealed that isolated bacteria belonged to the following genera: *Shewanella* (35.32%), *Proteus* (11.76%), *Paenibacillus* (11.76%), *Enterobacter* (11.76%), *Citrobacter* (11.76%), *Aeromonas* (5.88%), *Pseudomonas* (5.88%) and *Pantoea* (5.88%) (Table 1 and Fig. 2). The 1500‐bp‐long 16S rRNA gene containing nine hypervariable regions (V1–V9) (Chakravorty *et al*., 2007) was sequenced in single chain and, in all instances during phylogenetic analysis, the percentage similarity was ≥ 99%. Isolation of bacterial strains in the present work was performed under the conditions and culture medium explored, and the percentages observed only correspond to data observed in this work although other bacteria which could not grow under the explored conditions could exist in the same environment. There were no available data in literature to compare with the data obtained in this study, making this study potentially the first study to isolate and characterize bacterial isolates from carwash effluents.

**Table 1:** Characterization of isolates by isolate codes, sequence length, percentage similarity to closest matching strains and accession numbers

Isolate code	Sequence length (nt)	Closest match	% similarity	Accession number
BPS1	916	*Shewanella xiamenensis*	99	KX885451
BPS2	942	*Proteus penneri* strain wf‐4	99	KX885439
CAC11	928	*Aeromonas tecta* strain L47	100	KX885441
CAC12	826	*Paenibacillus lautus*	100	KX885440
CAC13	822	*Paenibacillus* sp. HF_07	100	KX885444
CAL2	948	*Shewanella* sp. LH8	100	KX885437
CAL3	974	*Proteus vulgaris* strain NWG20141026	100	KX885445
PCW5	868	*Shewanella* sp. JG353	100	KX885449
PCW6	815	*Citrobacter braakii* strain 425C3	100	KX885442
PCW7	931	*Citrobacter* sp. 29Kp3	100	KX885448
SAS10	935	*Shewanella putrefaciens* strain CZ‐BHG004	99	KX885452
SAS8	915	*Pseudomonas protegens*	100	KX885438
SAS9	904	*Pantoea* sp. 82353	99	KX885443
TCWPM1	847	*Shewanella* sp. JG353	100	KX885453
TCWPM2	871	*Shewanella* sp. UIWRF0468	99	KX885450
TCWPM3	894	*Enterobacter xiangfangensis* strain CDDS 11	100	KX885447
TCWPM4	887	*Enterobacter xiangfangensis* strain CDDS 11	99	KX885446

The phylogenetic tree diagram (Fig. 2) showed that isolates *Pseudomonas* sp. SAS8, *Paenibacillus* sp. CAC12 and *Paenibacillus* sp. CAC13 were distinctly different from the rest of the isolates, while isolates *Shewanella* sp. TCWPM1, *Shewanella* sp. TCWPM2, *Shewanella* sp. PCW5, *Shewanella* sp. BPS1, *Shewanella* sp. SAS10 and *Shewanella* sp. CAL2 formed were closely related.

### Screening for enzyme production (lipase and cellulase) and hydrocarbon utilization

Four isolates, three from carwash CAC namely *Aeromonas* sp. CAC11, *Paenibacillus* sp. CAC12 and *Paenibacillus* sp. CAC13 and one from carwash PCW namely *Citrobacter* sp. PCW7, were able to utilize hydrocarbon as a carbon source during preliminary screening (Table 2). Further, *Paenibacillus* sp. CAC12 and *Paenibacillus* sp. CAC13 were the only isolates to utilize the CMC sodium salt as a carbon source, indicating their ability to produce cellulase. Screening for lipase production showed that only isolate *Proteus* sp. BPS2, *Pseudomonas* sp. SAS8 and *Proteus* sp. CAL3 could hydrolyse olive oil.

**Table 2:** Screening results for hydrocarbon degradation, cellulase and lipase production

Isolate	Substrate		
	Hydrocarbon	CMC salt	Olive oil
BPS1	**−**	**−**	**−**
BPS2	**−**	**−**	+
CAC11	+	**−**	**−**
CAC12	+	+	**−**
CAC13	+	+	**−**
CAL2	**−**	**−**	**−**
CAL3	**−**	**−**	+
PCW5	**−**	**−**	**−**
PCW6	**−**	**−**	**−**
PCW7	+	**−**	**−**
SAS10	**−**	**−**	**−**
SAS8	**−**	**−**	+
SAS9	**−**	**−**	**−**
TCWPM1	**−**	**−**	**−**
TCWPM2	**−**	**−**	**−**
TCWPM3	**−**	**−**	**−**
TCWPM4	**−**	**−**	**−**

There are environments which, consequent to their substrate profiles, are better suited for the growth and proliferation of microorganisms producing certain kinds of enzymes. For example, a municipal solid waste site is rich in lignocellulosic materials, thus providing a suitable environment for cellulase‐producing organisms (Ali *et al*., 2013), while oil mill soils are likely to be endowed with lipase‐producing organisms (Ramesh *et al*., 2014). However, extreme environments are known to support microbial life forms with highly flexible metabolic pathways (Stathopoulou *et al*., 2013; Dalmaso *et al*., 2015). It is this flexibility, which causes extremophilic microorganisms to be the best known producers of biotechnologically important molecules/products (Bisht and Panda, 2011; Bull, 2011; Azua‐Bustos and González‐Silva, 2014). As in this study, production of cellulase by *Paenibacillus* sp. has previously been confirmed (Pandey *et al*., 2013). Similarly, production of lipase by *Pseudomonas* and *Proteus* has been confirmed in previous studies (Golani *et al*., 2016; Kumar *et al*., 2016). What has not been reported, however, is the occurrence of enzyme‐producing microorganisms from carwash effluent samples. In the wake of growing demand for enzymes with improved catalytic performance and tolerance to process‐specific parameters (Guazzaroni *et al*., 2015), it is imperative that every kind of environment be surveyed for enzyme‐producing microbes.

### Hydrocarbon degradation assays

**Figure 3:**
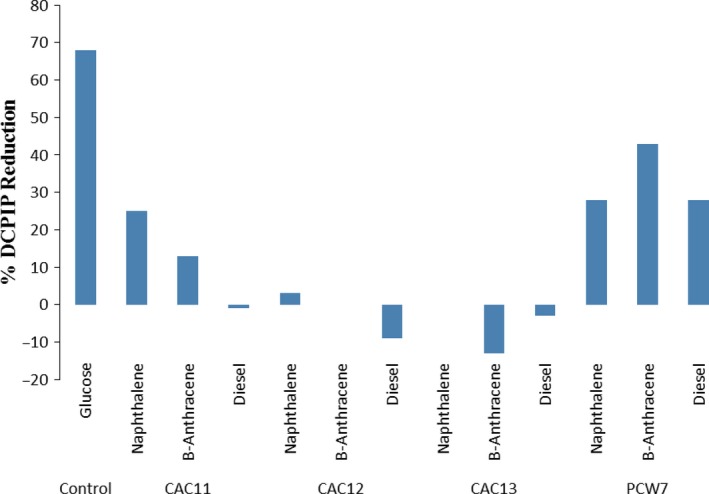
Percentage reduction in DCPIP during hydrolysis of hydrocarbons by four bacterial isolates.

Isolates *Aeromonas* sp. CAC11, *Paenibacillus* sp. CAC12, *Paenibacillus* sp. CAC13 and *Citrobacter* sp. PCW7 were further applied to hydrocarbon degradation assays using three classes of hydrocarbons namely benzanthracene, naphthalene and diesel fuel using 2,6‐dichlorophenolindolphenol (DCPIP) as the indicator. DCPIP is an electron acceptor that becomes reduced (decolourized) when redox reactions occur, in this case when NADH is converted to NAD^+^ during microbial degradation of hydrocarbons (Kubota *et al*., 2008; Bidoia *et al*., 2012). During the assay, the OD_600_ at 24 and 48 h was still almost similar to the OD_600_ at 0 h with, however, a sudden significant reduction in OD_600_ at 72 h. The results are presented in Fig. 3. Ionescu *et al*. (2015) also observed that bacterial cells needed about 4 days for the activation of the transcriptional regulation of metabolic operons involved in producing the necessary enzymes to metabolize an unusual substrate.

Isolate *Citrobacter* sp. PCW7 showed the highest potential of the four test isolates to metabolize all three hydrocarbon classes, with the highest activity against benzanthracene (45% DCPIP reduction). Next was isolate *Aeromonas* sp. CAC11, which showed 25% reduction in DCPIP when metabolizing naphthalene and 15% when metabolizing benzanthracene. Isolates *Paenibacillus* sp. CAC12 and *Paenibacillus* sp. CAC13 did not result in any significant DCPIP reduction. High levels of polycyclic aromatic hydrocarbons in environmental media have generated considerable interest from a toxicological standpoint (Al‐Daghri *et al*., 2014). Benzanthracene, for instance, is an environmentally recalcitrant pollutant, which is classified as a group 2A carcinogen by the International Agency for Research on cancer (Kunihiro *et al*., 2013). Besides naphthalene and benzanthracene, diesel fuel contains alkylbenzenes, toluene, naphthalenes and polycyclic aromatic hydrocarbons (Irwin *et al*., 1997), constituents which have already been implicated in asthma and cancer cases (Al‐Daghri *et al*., 2013). As such, it is of public and environmental health interest to find microorganisms which can biodegrade such hydrocarbons to rid or lessen the danger that they pose to public health, more so if such organisms are indigenous to the polluted environments. Isolates *Citrobacter* sp. PCW7 and *Aeromonas* sp. CAC11 showed much promise as potential microorganisms that can be used for the bioremediation of environments polluted by high molecular weight hydrocarbons. Other studies (Mandri and Lin, 2007; Affandi *et al*., 2014) have also proved the high diesel oil and grease degradation potentials of *Citrobacter* (*C. freundii*) and *Aeromonas* (*A. hydrophila*), which were, however, isolated from hydrocarbon‐contaminated environments, as in the present study. Most other studies, which have assessed the hydrocarbon degradation potentials of *Aeromonas* and *Shewanella*, used test strains that were isolated from hydrocarbon‐contaminated environmental media (Martín‐Gil *et al*., 2004; Ben Said *et al*., 2008; Hamzah *et al*., 2010). However, the study of Deppe *et al*. (2005) showed that *Shewanella* sp. isolated from a hydrocarbon‐free environment were unable to degrade crude oil except when it was applied in consortia with other bacteria. This shows that these bacterial strains have evolved in the explored environments to degrade hydrocarbons. Bacteria of the genera *Alcaligenes*,* Stenotrophomonas*,* Sphingomonas* and *Pseudomonas* have also been documented to biodegrade benzanthracene (Kunihiro *et al*., 2013), although the metabolites were not documented, as was the case in this study.

Elsewhere, Bonfá *et al*. (2011) described the isolation of aromatic hydrocarbon‐degrading haloarchaea in hypersaline environments. As in this particular study, the hypersaline environment from which they isolated the organisms was synthetic, created as a result of the discharge of ‘produced’ hypersaline water from gas and oil extraction activities. One point comes to the fore; that environmental pollution of whatever nature may indeed eliminate most forms of life (including some microorganisms), but it will always result in selective proliferation of other groups of microorganisms that will utilize the pollutant as a food source or a means to it. It is that particular ability to not only survive but thrive in polluted/adverse environments that makes such microorganisms potential bioresources (or sources thereof) for the ecological recovery of polluted sites. Further to, the existence of polyaromatic hydrocarbon‐degrading bacteria like *Citrobacter* sp. PCW7 and *Aeromonas* sp. CAC11 in the natural environment could be exerting a protective effect on higher organisms by continuously removing potentially harmful hydrocarbons from the environment.

### Secondary metabolite mapping using GC‐MS and UHPLC‐MS

**Figure 4:**
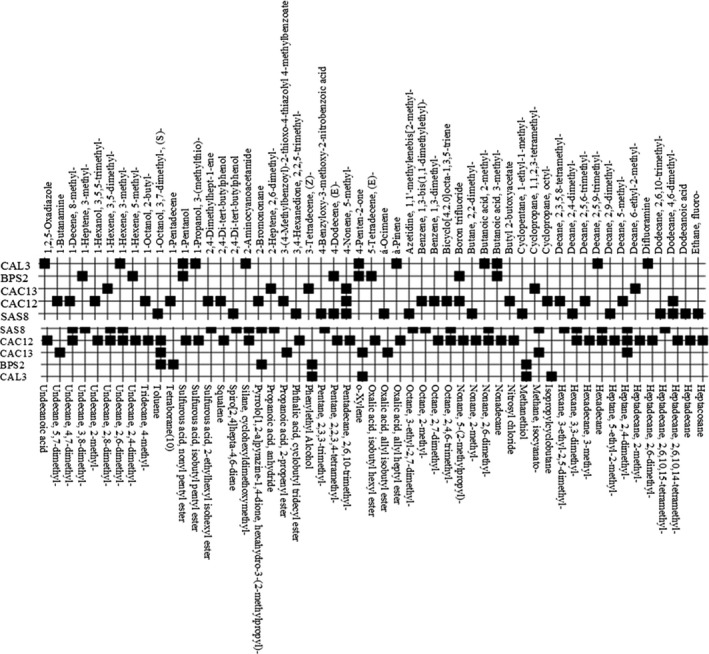
A map of secondary metabolites produced by bacterial isolates from carwash effluents as detected by GC‐MS.

GC‐MS secondary metabolite elucidation was able to identify 107 different compounds produced by five bacterial isolates, which had tested positive when screened for production of either lipase or cellulase. These were *Proteus* sp. BPS2, *Paenibacillus* sp. CAC12, *Pseudomonas* sp. SAS8, *Proteus* sp. CAL3 and *Paenibacillus* sp. CAC13. The compounds were identified by comparison of their mass spectra with the NIST library based on their molecular weight and retention time. To ensure a higher degree of accuracy, the minimum similarity match cut‐off for compound identification was pegged at 700. The compounds were configured into a map (Fig. 4) through multivariate analysis (seriation) using the paleontological statistical software (PAST3.13, Hammer 1999–2016). Looking at the number of compounds that were produced, it would have been virtually impossible to discuss each of them in detail; hence, the data were also subjected to principal component analysis (PCA) using SIMCA (SIMCA 14.0 Ink, UMETRICS Company 1998–2015, Malmö, Sweden) and the compounds which fell off the nucleus were chosen for further analysis and discussion. These are presented in Table 3.

**Table 3:** Description of structure and uses of some bacterial secondary metabolites identified by GC‐MS

Isolate	Compound name and chemical structure	Known/potential applications	References
CAL3	1,2,5‐oxadiazole 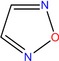	Major scaffold in the development of potential anticancer agents	(Boiani *et al*., 2001; Kumar *et al*., 2014)
CAC12	Spiro[2,4]hepta‐4,6‐diene 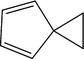	Used in synthesis of biologically active compounds ranging from pesticides to therapeutic drugs to enzymes	(Menchikov and Nefedov, 2016)
SAS8	α‐pinene 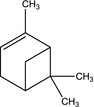	Major component of some therapeutic plant oils known for antibacterial activity against any bacterial species	(Baik *et al*., 2008; Hernández *et al*., 2013)
CAL3, BPS2	Phenylethyl alcohol 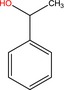	Antimicrobial preservative in pharmaceutical products like nasal sprays. Used to make selective agar for growth of Gram‐positive microbes	(Brewer and Lilley, 2011; Reza *et al*., 2015)
CAC12, SAS8	Silane, Cyclohexyldimethoxymethyl‐ 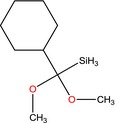	Used to mediate interfacial bonding in mineral reinforced dental polymeric composites	(Antonucci *et al*., 2005)
CAC12, CAC13	Cyclopentane 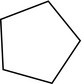	Cyclopentane fatty acids have potential antifungal activity against *Candida albicans*,* C. glabrata* and *Pythium ultimum*	(Pohl *et al*., 2011)
SAS8, BPS2	Pyrrolo[1,2a]pyrazine‐1, 4‐dione, hexahydro‐3‐(2‐methylpropyl)‐ 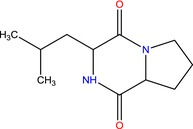	Exhibits antibacterial, antifungal, nematicidal and anticancer properties. Microbial and plant extracts containing this compound are commonly used as broad spectrum antibiotics	(Moniruzzaman *et al*., 2015; Mohan *et al*., 2016; Ser *et al*., 2016; Sharma *et al*., 2016)
CAC12	3‐(4‐Methylbenzoyl)‐2‐thioxo‐4‐thiazolyl 4‐methylbenzoate 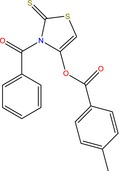	Uses include inhibition of tau fibril formation and thioflavin T binding, inhibition of *Bacillus subtilis* Sfp phosphopantetheinyl transferase (PPTase), and identification of small molecule inhibitors of *Plasmodium falciparum* Glucose‐6‐phosphate dehydrogenase via a fluorescence intensity assay	(NCBI, 2016)

Isolate *Paenibacillus* sp. CAC12 produced the most number of biotechnologically important molecules. These included cyclopentane, silane (cyclohexyldimethoxymethyl‐), spiro[2,4]hepta‐4,6‐diene and 3‐(4‐methylbenzoyl)‐2‐thioxo‐4‐thiazolyl 4‐methylbenzoate. With the exception of silane, which is used as an important component of polymeric substances for filling dental cavities (Antonucci *et al*., 2005), all the other metabolites are known bioactive compounds exhibiting either antifungal or antibacterial activities. While no studies have previously reported production of any of these compounds by any member of the genus *Paenibacillus*, Pohl *et al*. (2011) reported microbial production of cyclopentane in the form of a C17 cyclopentane fatty acid from *Pseudomonas*. Zhang *et al*. (2015) have, however, previously extracted cyclopentane from *Cunninghamia lanceolate* wood extracts known for their rich drug compositions, using GC‐MS. Peng *et al*. (2013) also extracted it from *Illicium verum* fruit, a medicinal plant that is known for having many active ingredients. As according to Zhang *et al*. (2015), the output of bioactive ingredients from herbaceous plants is too little to meet market demand, biotechnological approaches targeting enzyme‐producing microorganisms or their genes can be employed to amplify production of such biotechnologically important molecules.

Production of the compound 3‐(4‐methylbenzoyl)‐2‐thioxo‐4‐thiazolyl 4‐methylbenzoate was of particular interest in this study as no research records could be found of its production by any microorganism. The only information that could be accessed on this compound was about its multiple uses which ranged from inhibition of tau fibril formation to inhibition of *Bacillus subtilis* virulence mechanisms to inhibition of parasitic mechanisms of the malaria parasite *Plasmodium falciparum* (NCBI, 2016). Formation of tau fibrils has been linked to Alzheimer's disease, which is one of the most common causes of dementia in adults (Morozova *et al*., 2013; Dinkel *et al*., 2015). Clearly, this is a very important compound with potential for many other applications, and further research on its production and applications is highly recommended.

A glance at Table 3 also shows that most of the compounds produced by the isolates are bioactive ingredients of commercial antimicrobials or plant extracts. The compound pyrrolo[1,2a]pyrazine‐1, 4‐dione, hexahydro‐3‐(2‐methylpropyl), for instance, has antifungal, antibacterial, nematicidal and anticancer activities. Its antimicrobial profile suggests a need by the producing isolates (*Pseudomonas* sp. SAS8 and *Proteus* sp. BPS2) to competitively suppress the growth of other competing microorganisms, which could be likely due to restricted resources in their environment. Hibbing *et al*. (2010) allude to the fact that in cases of stiff competition for resources, microbial communities could resort to production of colicins (bacteriocins targeting enteric bacteria) and/or antibiotics among other strategies of limiting the growth of competing species. Microorganisms, which produce anticancer drugs, deserve a much bigger attention given the growing global incidence of this disease, and the discomfort of the current chemotherapeutic methods of treating it.

Although a few selected compounds were given extensive analysis and discussion, a potentially high number of biotechnologically important molecules from the metabolite map (Fig. 4) remain unexplored. This opens up new avenues for further research to fully tap into these microbial bioresources. Also, while compound determination by both GC‐MS and LC‐MS was only qualitative, the presence of any of the identified compounds points to the presence of the encoding gene(s) within the bacterial isolates. Application of biotechnological techniques like gene cloning can therefore be used to amplify production of the compounds to commercial levels.

### Compounds identified by LC‐MS

**Figure 5:**
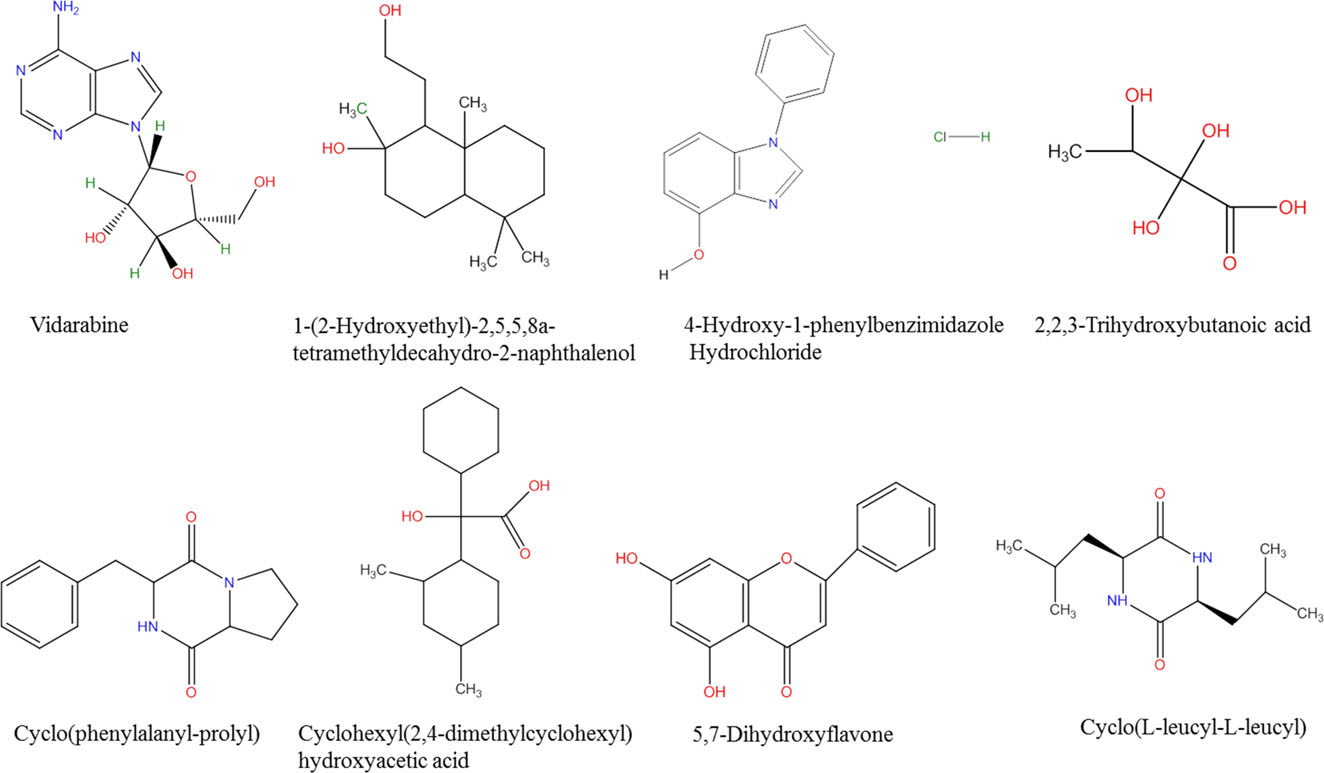
Structural elucidation of bacterial secondary metabolites identified using UHPLC‐MS.

Elucidation of microbial secondary metabolites was performed using the Bruker software. LC‐MS results were first subjected to principal component analysis (PCA), which helped in identifying those compounds which were significantly different from the rest. Structural elucidation of the compounds was performed using online libraries including KEGG, Pubchem and Chemspider. Principal component 1 (PC1) showed that isolates *Pseudomonas* sp. SAS8, *Paenibacillus* sp. CAC12 and *Paenibacillus* sp. CAC13 produced metabolites, which were significantly different from those of other isolates. PC2 further showed that while metabolites produced by isolates *Paenibacillus* sp. CAC12 and *Paenibacillus* sp. CAC13 were comparably similar, they were, however, different from metabolites produced by isolate *Pseudomonas* sp. SAS8. PCA loadings showed that all the isolates could produce the compounds cyclo(phenylalanyl‐prolyl), 2,2,3‐trihydroxybutanoic acid, 5,7‐dihydroxyflavone, cyclo(L‐leucyl‐L‐leucyl), vidarabine, 2‐(4‐hydroxyphenyl)ethyl tricosanoate and 1‐(2‐hydroxyethyl)‐2,5,5,8a‐tetramethyldecahydro‐2‐naphthalenol, while only isolate *Paenibacillus* sp. CAC12 and *Paenibacillus* sp. CAC13 could produce cyclohexyl(2,4‐dimethylcyclohexyl)hydroxyacetic acid. Similarly, the compound 4‐hydroxy‐1‐phenylbenzimidazole hydrochloride could only be produced by the isolate *Pseudomonas* sp. SAS8. The molecular structures of the compounds are shown in Fig. 5.

The compound 5,7‐dihydroxyflavone (chrysin), which was produced by all isolates, is a known natural flavonoid found in many plant extracts that are commonly used as wound‐healing, skin‐protective and anticancer medicines (Sathishkumar *et al*., 2015). Chrysin is also known for its anti‐inflammatory and antioxidation properties (Engelmann *et al*., 2005). The research of Kim *et al*. (2011) also found chrysin to be an effective inhibitor against hyperpigmentation by directly inhibiting the activity of adenylyl cyclase, a key enzyme involved in cAMP‐induced melanogenesis. Other researchers (Li *et al*., 2015) have proven that chrysin is an antihypertensive drug, which acts by inhibiting the proliferation and migration of pulmonary artery smooth muscle cells, together with the associated extracellular matrix components, principally, collagens. Given its important range of biological activities, microbial production of chrysin could be manipulated to significantly increase its production both as a therapeutic agent and also as a dietary supplement.

The compound 2‐(4‐hydroxyphenyl)ethyl tricosanoate, which was also identified in the crude extracts of all isolates, was previously isolated by Acevedo *et al*. (2000) as one of the components of the crude extracts of *Buddleja cordata* subsp. *cordata* known for antimycobacterial activity. In addition, the same compound is traditionally used in various other applications including treatment of diarrhoea, headaches and kidney ailments (Acevedo *et al*., 2000). Literature records of microbial production of 2‐(4‐hydroxyphenyl)ethyl tricosanoate are scarce, suggesting there could be many metabolites which are currently known to be produced by plants only when, in fact, they are produced by microorganisms also.

One of the identified compounds, cyclo(phenylalanyl‐prolyl), is a known cyclic dipeptide, which is used as a scaffold for drugs besides its use as an antiviral, antibiotic and antitumour drug (Wickrama Arachchilage *et al*., 2012). Microbial production of this dipeptide has previously been reported in a *Streptomyces* sp. isolated from marine sediments (Macherla *et al*., 2005). Vidarabine is yet another compound with antiviral properties that was identified in the crude extracts of all isolates. Vidarabine is a purine nucleotide analog that inhibits viral DNA synthesis and has previously been identified in the extracts of a Caribbean sponge *Cryptotethia crypta* (Suzuki *et al*., 2006). Literature is awash with evidence of production of vidarabine by marine bacteria and sponges (Sagar *et al*., 2010; Wang *et al*., 2013; Nadeem *et al*., 2016). To our knowledge, this is the first report of the production of vidarabine by bacteria isolated from a non‐marine environment. Of late, however, use of vidarabine as an antiviral agent has been declining owing to its neurotoxicity, which has prompted research for more effective and less toxic alternatives (Vajpayee and Malhotra, 2000). Other identified compounds including 1‐(2‐hydroxyethyl)‐2,5,5,8a‐tetramethyldecahydro‐2‐naphthalenol, 4‐hydroxy‐1‐phenylbenzimidazole hydrochloride, 2,2,3‐trihydroxybutanoic acid and cyclohexyl(2,4‐dimethylcyclohexyl)hydroxyacetic acid have no clear known uses, as does a host of other compounds which neither of the LC‐MS linked online libraries could detect. This shows that exploration of novel microbial metabolites still has a long way to go. Dalmaso *et al*. (2015) estimated that between 1 and 10% of prokaryotes have so far been described, and this equates more or less to microbial secondary metabolites.

Most of the metabolites identified in this study have previously been documented to have antibacterial, antifungal and/or anticancer properties, although this was not experimentally proven in the present study. This finding, however, was to be expected since currently, the most marketed antimicrobials and anticancer drugs are natural products of microbial origin (Shaaban *et al*., 2013). It follows that microbial populations in different ecological settings including natural and synthetic extreme environments still have a lot to offer in terms of bioresources. In a bid to increase the success rate in the identification of novel microbial secondary metabolites, the use of GC‐MS and UHPLC‐MS in chemical profiling of crude fermentation extracts can be a very useful tool for assessing the chemical novelty of the crude extracts by comparing the mass spectra to in‐built and online compound libraries such as Chemspider, Pubchem and KEGG.

## Conclusion

Synthetic extreme environments have been overlooked as potential sources of biotechnologically relevant microbiota. The outcome of this research indicates that some microbial metabolites which have previously been known to be produced only by microorganisms in natural extreme environments like the marine environment can also be produced by microorganisms in synthetic extreme environments like carwashes. Further, this research has revealed the immense bioresource potential of microorganisms inhabiting synthetic extreme environments, which harbour potential as agents of bioremediation and/or producers of biotechnologically relevant molecules including enzymes.

## Conflict of interest

The authors wish to declare no conflict of interests with respect to submission, review and potential publication of this manuscript.

## References

[mbt212602-bib-0001] Acevedo, L. , Martínez, E. , Castañeda, P. , Franzblau, S. , Timmermann, B.N. , Linares, E. , *et al* (2000) New phenylethanoids from *Buddleja cordata* subsp. cordata. Planta Med 66: 257–261. 10821053 10.1055/s-2000-8570

[mbt212602-bib-0002] Affandi, I.E. , Suratman, N.H. , Abdullah, S. , Ahmad, W.A. , and Zakaria, Z.A. (2014) Degradation of oil and grease from high‐strength industrial effluents using locally isolated aerobic biosurfactant‐producing bacteria. Int Biodeterior Biodegrad 95: 33–40.

[mbt212602-bib-0003] Akmal, M. (2009) Microbial biomass and bacterial community changes by Pb contamination in acidic soil. J Agric Biol Sci 1: 30–37.

[mbt212602-bib-0004] Al‐Daghri, N.M. , Alokail, M.S. , Abd‐Alrahman, S.H. , Draz, H.M. , Yakout, S.M. , and Clerici, M. (2013) Polycyclic aromatic hydrocarbon exposure and pediatric asthma in children: a case‐control study. Environ Heal A Glob Access Sci Source 12: 2–7. 10.1186/1476-069X-12-1 PMC3621697 23286340

[mbt212602-bib-0005] Al‐Daghri, N.M. , Alokail, M.S. , Abd‐Alrahman, S.H. , and Draz, H.M. (2014) Polycyclic aromatic hydrocarbon distribution in serum of Saudi children using HPLC‐FLD: marker elevations in children with asthma. Environ Sci Pollut Res Int 21: 12085–12090. 24923226 10.1007/s11356-014-3108-0

[mbt212602-bib-0006] Ali, J. , Ahmad, B. , Nigar, S. , Sadaf, S. , Shah, A. , Bashir, S. , *et al* (2013) Isolation and identification of cellulose degrading bacteria from municipal waste and their screening for potential antimicrobial activity. World Appl Sci J 27: 1420–1426.

[mbt212602-bib-0007] Antonucci, J.M. , Dickens, S.H. , Fowler, B.O. , and Xu, H.H.K. (2005) Chemistry of silanes: interfaces in dental polymers and composites. J Res Natl Inst Stand Technol 110: 541. 27308178 10.6028/jres.110.081 PMC4847576

[mbt212602-bib-0008] Azua‐Bustos, A. , and González‐Silva, C. (2014) Biotechnological applications derived from microorganisms of the Atacama Desert. Biomed Res Int 2014: 1–7. 10.1155/2014/909312 PMC4132489 25147824

[mbt212602-bib-0009] Baik, J.S. , Kim, S.‐S. , Lee, J.‐A. , Oh, T.‐H. , Kim, J.‐Y. , Lee, N.H. , and Hyun, C.‐G. (2008) Chemical composition and biological activities of essential oils extracted from Korean endemic citrus species. J Microbiol Biotechnol 18: 74–79. 18239420

[mbt212602-bib-0010] Bayat, Z. , Hassanshahian, M. , and Cappello, S. (2015) Immobilization of microbes for bioremediation of crude oil polluted environments : a mini review. Open Microbiol J 9: 48–54. 26668662 10.2174/1874285801509010048 PMC4676050

[mbt212602-bib-0011] Ben Said, O. , Goñi‐Urriza, M.S. , El Bour, M. , Dellali, M. , Aissa, P. , and Duran, R. (2008) Characterization of aerobic polycyclic aromatic hydrocarbon‐degrading bacteria from Bizerte lagoon sediments, Tunisia. J Appl Microbiol 104: 987–997. 17973912 10.1111/j.1365-2672.2007.03621.x

[mbt212602-bib-0012] Bidoia, E.D. , Montagnolli, R.N. , and Lopes, P.R.M. (2012) Microbial biodegradation potential of hydrocarbons evaluated by colorimetric technique: a case study. Appl Microbiol Microb Biotechnol 3: 1277–1288.

[mbt212602-bib-0013] Bisht, S.S. , and Panda, A.K. (2011) Biochemical characterization and 16S rRNA sequencing of few lipase‐producing thermophilic bacteria from Taptapani hot water spring, Orissa, India. Biotechnol Res Int 2011: 452710. 21541249 10.4061/2011/452710 PMC3085305

[mbt212602-bib-0014] Boiani, M. , Cerecetto, H. , and González, M. (2001) 1, 2, 5‐Oxadiazole N‐oxide derivatives as potential anti‐cancer agents: synthesis and biological evaluation. Part IV. Eur J Med Chem 36: 771–782. 11738485 10.1016/s0223-5234(01)01265-x

[mbt212602-bib-0015] Bonfá, M.R.L. , Grossman, M.J. , Mellado, E. , and Durrant, L.R. (2011) Biodegradation of aromatic hydrocarbons by Haloarchaea and their use for the reduction of the chemical oxygen demand of hypersaline petroleum produced water. Chemosphere 84: 1671–1676. 21621813 10.1016/j.chemosphere.2011.05.005

[mbt212602-bib-0016] Brewer, J.H. , and Lilley, B.D. (2011) Phenylethyl Alcohol Agar. Microbe Libr 5: 1–8.

[mbt212602-bib-0017] Bull, A.T. (2011) Bioprospecting Among the Actinobacteria of Extreme Environments. University of Kent, Canterbury, UK.

[mbt212602-bib-0018] Cavicchioli, R. , Siddiqui, K.S. , Andrews, D. , and Sowers, K.R. (2002) Low‐temperature extremophiles and their applications. Curr Opin Biotechnol 13: 253–261. 12180102 10.1016/s0958-1669(02)00317-8

[mbt212602-bib-0019] Chakravorty, S. , Helb, D. , Burday, M. , and Connell, N. (2007) A detailed analysis of 16S ribosomal RNA gene segments for the diagnosis of pathogenic bacteria. J Microbiol Methods 69: 330–339. 17391789 10.1016/j.mimet.2007.02.005 PMC2562909

[mbt212602-bib-0020] Chronakova, A. , Kristufek, V. , Tichy, M. , and Elhottova, D. (2010) Biodiversity of streptomycetes isolated from a succession sequence at a post‐mining site and their evidence in Miocene lacustrine sediment. Microbiol Res 165: 594–608. 20015625 10.1016/j.micres.2009.10.002

[mbt212602-bib-0021] Dalmaso, G.Z.L. , Ferreira, D. , and Vermelho, A.B. (2015) Marine extremophiles a source of hydrolases for biotechnological applications. Mar Drugs 13: 1925–1965. 25854643 10.3390/md13041925 PMC4413194

[mbt212602-bib-0022] Demirjian, D.C. , Morís‐varas, F. , and Cassidy, C.S. (2001) Enzymes from extremophiles. Curr Opin Chem Biol 5: 144–151. 11282340 10.1016/s1367-5931(00)00183-6

[mbt212602-bib-0023] Deppe, U. , Richnow, H.H. , Michaelis, W. , and Antranikian, G. (2005) Degradation of crude oil by an arctic microbial consortium. Extremophiles 9: 461–470. 15999222 10.1007/s00792-005-0463-2

[mbt212602-bib-0024] Dinkel, P.D. , Holden, M.R. , Matin, N. , and Margittai, M. (2015) RNA binds to tau fibrils and sustains template‐assisted growth. Biochemistry 54: 4731–4740. 26177386 10.1021/acs.biochem.5b00453 PMC4526887

[mbt212602-bib-0025] Enache, M. , and Kamekura, M. (2010) Hydrolytic enzymes of halophilic microorganisms and their economic values. Rom J Biochem 47: 47–59.

[mbt212602-bib-0026] Engelmann, M.D. , Hutcheson, R. , and Cheng, I.F. (2005) Stability of ferric complexes with 3‐hydroxyflavone (flavonol), 5,7‐dihydroxyflavone (chrysin), and 3’,4’‐dihydroxyflavone. J Agric Food Chem 53: 2953–2960. 15826045 10.1021/jf048298q

[mbt212602-bib-0027] Ettoumi, B. , Chouchane, H. , Guesmi, A. , Mahjoubi, M. , Brusetti, L. , Neifar, M. , *et al* (2016) Diversity, ecological distribution and biotechnological potential of Actinobacteria inhabiting seamounts and non‐seamounts in the Tyrrhenian Sea. Microbiol Res 186–187: 71–80. 10.1016/j.micres.2016.03.006 27242145

[mbt212602-bib-0028] Golani, M. , Hajela, K. , and Pandey, G.P. (2016) Screening. Identification, characterization and production of bacterial lipase from oil spilled soil. Asian J Pharm Clin Res 5: 745–763.

[mbt212602-bib-0029] Guazzaroni, M.E. , Silva‐Rocha, R. , and Ward, R.J. (2015) Synthetic biology approaches to improve biocatalyst identification in metagenomic library screening. Microb Biotechnol 8: 52–64. 25123225 10.1111/1751-7915.12146 PMC4321373

[mbt212602-bib-0030] Hamzah, A. , Rabu, A. , Azmy, R.F.H.R. , and Yussoff, N.A. (2010) Isolation and characterization of bacteria degrading Sumandak and South Angsi oils. Sains Malaysiana 39: 161–168.

[mbt212602-bib-0031] Hernández, V. , Mora, F. , Araque, M. , De Montijo, S. , Rojas, L. , Meléndez, P. , and De Tommasi, N. (2013) Chemical composition and antibacterial activity of *Astronium graveolens* JACQ essential oil. Rev Latinoam Quim 41: 89–94.

[mbt212602-bib-0032] Hibbing, M.E. , Fuqua, C. , Parsek, M.R. , and Peterson, S.B. (2010) Bacterial competition: surviving and thriving in the microbial jungle. Natl Rev Microbiol 8: 15–25. 10.1038/nrmicro2259 PMC2879262 19946288

[mbt212602-bib-0033] Huber, H. , and Stetter, K.O. (1998) Hyperthermophiles and their possible potential in biotechnology. J Biotechnol 64: 39–52.

[mbt212602-bib-0034] Ionescu, R. , Măruţescu, L. , Tănase, A.‐M. , Chiciudean, I. , Csutak, O. , Pelinescu, D. , *et al* (2015) Flow cytometry based method for evaluation of biodegradative potential of *Pseudomonas fluorescens* . Agric Agric Sci Procedia 6: 567–578.

[mbt212602-bib-0035] Irwin, R. , Van Mouwerik, M. , Stevens, L. , Seese, M. and Basham, W. (1997) Environmental Contaminants Encyclopedia: Diesel Oil Entry. Fort Collins, CO: National Park Service, Water Resources Divisions, Water Operations Branch.

[mbt212602-bib-0036] Jami, M. , Ghanbari, M. , Kneifel, W. , and Domig, K.J. (2015) Phylogenetic diversity and biological activity of culturable Actinobacteria isolated from freshwater fish gut microbiota. Microbiol Res 175: 6–15. 25662514 10.1016/j.micres.2015.01.009

[mbt212602-bib-0037] Kim, D.C. , Rho, S.H. , Shin, J.C. , Park, H.H. , and Kim, D. (2011) Inhibition of melanogenesis by 5,7‐dihydroxyflavone (chrysin) via blocking adenylyl cyclase activity. Biochem Biophys Res Commun 411: 121–125. 21723261 10.1016/j.bbrc.2011.06.108

[mbt212602-bib-0038] Kuang, W. , Li, J. , Zhang, S. , and Long, L. (2015) Diversity and distribution of Actinobacteria associated with reef coral *Porites lutea* . Front Microbiol 6: 1–13. 26539166 10.3389/fmicb.2015.01094 PMC4612714

[mbt212602-bib-0039] Kubota, K. , Koma, D. , Matsumiya, Y. , Chung, S.Y. , and Kubo, M. (2008) Phylogenetic analysis of long‐chain hydrocarbon‐degrading bacteria and evaluation of their hydrocarbon‐degradation by the 2,6‐DCPIP assay. Biodegradation 19: 749–757. 18283542 10.1007/s10532-008-9179-1

[mbt212602-bib-0040] Kumar, D. , Kumar, L. , Nagar, S. , Raina, C. , Parshad, R. , and Gupta, V.K. (2012) Screening, isolation and production of lipase/esterase producing Bacillus sp. strain DVL2 and its potential evaluation in esterification and resolution reactions. Arch Appl Sci Res 4: 1763–1770.

[mbt212602-bib-0041] Kumar, A. , Ito, A. , Takemoto, M. , Yoshida, M. , and Zhang, K.Y.J. (2014) Identification of 1,2,5‐oxadiazoles as a new class of SENP2 inhibitors using structure based virtual screening. J Chem Inf Model 54: 870–880. 24512059 10.1021/ci4007134

[mbt212602-bib-0042] Kumar, A. , Dhar, K. , Kanwar, S.S. , and Arora, P.K. (2016) Lipase catalysis in organic solvents: advantages and applications. Biol Proced Online 18: 2. 26766927 10.1186/s12575-016-0033-2 PMC4711063

[mbt212602-bib-0043] Kunihiro, M. , Ozeki, Y. , Nogi, Y. , Hamamura, N. , and Kanaly, R.A. (2013) Benz[a]anthracene biotransformation and production of ring fission products by Sphingobium sp. strain KK22. Appl Environ Microbiol 79: 4410–4420. 23686261 10.1128/AEM.01129-13 PMC3697515

[mbt212602-bib-0044] Li, X.‐W. , Wang, X.‐M. , Li, S. , and Yang, J.‐R. (2015) Effects of chrysin (5,7‐dihydroxyflavone) on vascular remodeling in hypoxia‐induced pulmonary hypertension in rats. Chin Med 10: 4. 25722740 10.1186/s13020-015-0032-2 PMC4341233

[mbt212602-bib-0045] Macherla, V.R. , Liu, J. , Bellows, C. , Teisan, S. , Nicholson, B. , Lam, K.S. , and Potts, B.C.M. (2005) Glaciapyrroles A, B, and C, pyrrolosesquiterpenes from a Streptomyces sp. isolated from an Alaskan marine sediment. J Nat Prod 68: 780–783. 15921430 10.1021/np049597c

[mbt212602-bib-0046] Maes, S. , Props, R. , Fitts, P. , Smet, R.De. , Vilchez‐vargas, R. , Vital, M. , *et al* (2016) Platinum recovery from synthetic extreme environments by halophilic bacteria. Environ Sci Technol 50: 2619–2626. 26854514 10.1021/acs.est.5b05355

[mbt212602-bib-0047] Mandri, T. , and Lin, J. (2007) Isolation and characterization of engine oil degrading indigenous microrganisms in Kwazulu‐Natal, South Africa. Afr J Biotechnol 6: 23–27.

[mbt212602-bib-0048] Markowicz, A. , Cyco, M. , and Piotrowska‐Seget, Z. (2016) Microbial community structure and diversity in long‐term hydrocarbon and heavy metal contaminated soils. Int J Environ Res 10: 321–332.

[mbt212602-bib-0049] Martín‐Gil, J. , Ramos‐Sánchez, M.C. , and Martín‐Gil, F.J. (2004) Shewanella putrefaciens in a fuel‐in‐water emulsion from the Prestige oil spill. *Antonie van Leeuwenhoek* . Int J Gen Mol Microbiol 86: 283–285. 10.1023/B:ANTO.0000047939.49597.eb 15539931

[mbt212602-bib-0050] Menchikov, L.G. , and Nefedov, O.M. (2016) Spiro[2.4]hepta‐4,6‐dienes: synthesis and application in organic synthesis. Russ Chem Rev 85: 205–225.

[mbt212602-bib-0051] Mohan, G. , Thangappanpillai, A.K. , and Ramasamy, B. (2016) Antimicrobial activities of secondary metabolites and phylogenetic study of sponge endosymbiotic bacteria, Bacillus sp. at Agatti Island, Lakshadweep Archipelago. Biotechnol Rep 11: 44–52. 10.1016/j.btre.2016.06.001 PMC5042297 28352539

[mbt212602-bib-0052] Moniruzzaman, S. , Haque, A. , Khatun, R. , and Yaakob, Z. (2015) Gas chromatography mass spectrometry analysis and in vitro antibacterial activity of essential oil from *Trigonella foenum‐graecum* . Asian Pac J Trop Biomed 5: 1033–1036.

[mbt212602-bib-0053] Morozova, O.A. , March, Z.M. , Robinson, A.S. , and Colby, D.W. (2013) Conformational features of tau fibrils from Alzheimer's disease brain are faithfully propagated by unmodified recombinant protein. Biochemistry 52: 6960–6967. 24033133 10.1021/bi400866w PMC4142060

[mbt212602-bib-0054] Nadeem, F. , Oves, M. , Qari, H. , and Ismail, I.M. (2016) Red sea microbial diversity for antimicrobial and anticancer agents. J Mol Biomark Diagn 7: 1–14.

[mbt212602-bib-0055] NCBI (2016) National Center for Biotechnology Information. PubChem Compound Database; CID=576784. Ncbi.

[mbt212602-bib-0056] Oliveira, N.C.De. , Rodrigues, A.A. , Alves, M.I.R. , Filho, N.R.A. , Sadoyama, G. , and Vieira, J.D.G. (2012) Endophytic bacteria with potential for bioremediation of petroleum hydrocarbons and derivatives. Afr J Biotechnol 11: 2977–2984.

[mbt212602-bib-0057] Pandey, S. , Singh, S. , Yadav, A.N. , Nain, L. , and Saxena, A.K. (2013) Phylogenetic diversity and characterization of novel and efficient cellulase producing bacterial isolates from various extreme environments. Biosci Biotechnol Biochem 77: 1474–1480. 23832366 10.1271/bbb.130121

[mbt212602-bib-0058] Peng, W. , Lin, Z. , Chang, J. , Gu, F. , and Zhu, X. (2013) Biomedical molecular characteristics of YBSJ extractives from *Illicium verum* fruit. Biotechnol Biotechnol Equip 27: 4311–4316.

[mbt212602-bib-0059] Perks, B. (2011) Extreme potential. Chem World 48–51.

[mbt212602-bib-0060] Pohl, C.H. , Kock, J.L.F. and Thibane, V.S. (2011) Antifungal free fatty acids: A Review. University of the Free State, Bloemfontein, South Africa.

[mbt212602-bib-0061] Ramesh, S. , Kumar, R. , Devi, R.A. , and Balakrishnan, K. (2014) Isolation of a lipase producing bacteria for enzyme synthesis in shake flask cultivation. Int J Curr Microbiol Appl Sci 3: 712–719.

[mbt212602-bib-0062] Rampelotto, P.H. (2013) Extremophiles and extreme environments. Life 3: 482–485. 25369817 10.3390/life3030482 PMC4187170

[mbt212602-bib-0063] Reed, C.J. , Lewis, H. , Trejo, E. , Winston, V. , and Evilia, C. (2013) Protein adaptations in archaeal extremophiles. Archaea 2013: 1–14. 10.1155/2013/373275 PMC3787623 24151449

[mbt212602-bib-0064] Reza, H. , Fereshteh, N. , and Saman, A.N. (2015) Determination of phenylethyl alcohol by reversed‐phase high‐performance liquid chromatography (RP‐HPLC) in Budesonide nasal spray. Afr J Pure Appl Chem 9: 81–90.

[mbt212602-bib-0065] Sagar, S. , Kaur, M. , and Minneman, K.P. (2010) Antiviral lead compounds from marine sponges. Mar Drugs 8: 2619–2638. 21116410 10.3390/md8102619 PMC2992996

[mbt212602-bib-0066] Sathishkumar, G. , Bharti, R. , Jha, P.K. , Selvakumar, M. , Dey, G. , Jha, R. , *et al* (2015) Dietary flavone chrysin (5,7‐dihydroxyflavone ChR) functionalized highly‐stable metal nanoformulations for improved anticancer applications. RSC Adv 5: 89869–89878.

[mbt212602-bib-0067] Selbmann, L. , Egidi, E. , Isola, D. , Onofri, S. , Zucconi, L. , de Hoog, G.S. , *et al* (2013) Biodiversity, evolution and adaptation of fungi in extreme environments. Plant Biosyst – An Int J Deal with all Asp Plant Biol 147: 237–246.

[mbt212602-bib-0068] Ser, H.‐L. , Palanisamy, U.D. , Yin, W.‐F. , Chan, K.‐G. , Goh, B.‐H. and Lee, L.‐H. (2016) *Streptomyces malaysiense* sp. nov.: a novel Malaysian mangrove soil actinobacterium with antioxidative activity and cytotoxic potential against human cancer cell lines. Sci Rep 6, 24247. 27072394 10.1038/srep24247 PMC4829849

[mbt212602-bib-0069] Seufferheld, M.J. , Alvarez, M. , and Farias, M.E. (2008) Role of polyphosphates in microbial adaptation to extreme environments. Appl Environ Microbiol 74: 5867–5874. 18708516 10.1128/AEM.00501-08 PMC2565944

[mbt212602-bib-0070] Shaaban, M. , Abdel‐Razik, A.S. , Abdel‐Aziz, M.S. , Abou Zied, A. , and Fadel, M. (2013) Bioactive secondary metabolites from marine *Streptomyces albogriseolus* isolated from Red Sea coast. J Appl Sci Res 9: 996–1003.

[mbt212602-bib-0071] Sharma, P. , Kalita, M.C. , and Thakur, D. (2016) Broad spectrum antimicrobial activity of forest‐derived soil actinomycete, Nocardia sp. PB‐52. Front Microbiol 7: 1–17. 27047463 10.3389/fmicb.2016.00347 PMC4796592

[mbt212602-bib-0072] Stathopoulou, P.M. , Savvides, A.L. , Karagouni, A.D. , and Hatzinikolaou, D.G. (2013) Unraveling the lipolytic activity of thermophilic bacteria isolated from a volcanic environment unraveling the lipolytic activity of thermophilic bacteria isolated from a volcanic environment. Biomed Res Int 2013: 1–13. 10.1155/2013/703130 PMC3662197 23738330

[mbt212602-bib-0073] Sutton, N.B. , Maphosa, F. , Morillo, J.A. , Al‐Soud, W.A. , Langenhoff, A.A.M. , Grotenhuis, T. , *et al* (2013) Impact of long‐term diesel contamination on soil microbial community structure. Appl Environ Microbiol 79: 619–630. 23144139 10.1128/AEM.02747-12 PMC3553749

[mbt212602-bib-0074] Suzuki, M. , Okuda, T. , and Shiraki, K. (2006) Synergistic antiviral activity of acyclovir and vidarabine against herpes simplex virus types 1 and 2 and varicella‐zoster virus. Antiviral Res 72: 157–161. 16797734 10.1016/j.antiviral.2006.05.001

[mbt212602-bib-0750] Tamura, K. , Stecher, G. , Peterson, D. , Filipski, A. , and Kumar, S. (2013) MEGA6: Molecular Evolutionary Genetics Analysis Version 6.0. Mol Biol Evol 30: 2725–2729. 24132122 10.1093/molbev/mst197 PMC3840312

[mbt212602-bib-0075] Tekere, M. , Sibanda, T. , and Maphangwa, K.W. (2016) An assessment of the physicochemical properties and toxicity potential of carwash effluents from professional carwash outlets in Gauteng Province, South Africa. Environ Sci Pollut Res 23: 11876–11884. 10.1007/s11356-016-6370-5 26957430

[mbt212602-bib-0076] Um, Y. , Chang, M.W. , and Holoman, T.P. (2010) A simple and effective plating method to screen polycyclic aromatic hydrocarbon‐degrading bacteria under various redox conditions. Appl Microbiol Biotechnol 88: 291–297. 20645084 10.1007/s00253-010-2761-6

[mbt212602-bib-0077] Vajpayee, M. , and Malhotra, N. (2000) Antiviral drugs against herpes infections. Indian J od Pharmacol 32: 330–338.

[mbt212602-bib-0078] Wang, Y. , Shi, J. , Wang, H. , Lin, Q. , Chen, X. , and Chen, Y. (2007) The influence of soil heavy metals pollution on soil microbial biomass, enzyme activity, and community composition near a copper smelter. Ecotoxicol Environ Saf 67: 75–81. 16828162 10.1016/j.ecoenv.2006.03.007

[mbt212602-bib-0079] Wang, R. , Billone, P.S. , and Mullett, W.M. (2013) Nanomedicine in action: an overview of cancer nanomedicine on the market and in clinical trials. J Nanomater 2013: 421–428.

[mbt212602-bib-0080] Wickrama Arachchilage, A.P. , Wang, F. , Feyer, V. , Plekan, O. , and Prince, K.C. (2012) Photoelectron spectra and structures of three cyclic dipeptides: PhePhe, TyrPro, and HisGly. J Chem Phys 136: 124301–124308. 22462851 10.1063/1.3693763

[mbt212602-bib-0081] Zhang, X. , Huang, K. , Ye, Y. , Shi, J. , and Zhang, Z. (2015) Biomedical molecular of woody extractives of *Cunninghamia lanceolata* biomass. Pak J Pharm Sci 28: 761–764. 25796151

